# Diurnal variation and practice effects in saccade task performance

**DOI:** 10.1007/s00221-025-07131-7

**Published:** 2025-07-23

**Authors:** Thomas Karantinos, Evi Kotsiou, Panagiota Drouza, Asimakis Mantas, Andrew J. Anderson, Christoph Klein, Nikolaos Smyrnis

**Affiliations:** 1 Laboratory of Cognitive Neuroscience and sensorimotor control, Neurosciences and Precision Medicine Research Institute “COSTAS STEFANIS”, University Mental Health, Athens, Greece; 2 2nd Department of Psychiatry, Medical School, National and Kapodistrian University of Athens, University General Hospital “ATTIKON”, 1 Rimini St., Athens, Greece; 3 Department of Optometry and Vision Sciences, The University of Melbourne, Melbourne, Australia; 4 Department of Child and Adolescent Psychiatry, University of Freiburg, Freiburg, Germany; 5 Department of Child and Adolescent Psychiatry, Medical Faculty, University of Cologne, Cologne, Germany

**Keywords:** Visually guided saccade, Gap saccade, Antisaccade, Countermanding saccade, Oculomotor tasks, Saccadic latency

## Abstract

Saccadic eye movement tasks have been widely used as a probe for measuring cognitive functions in healthy humans as well as in patients with neurological and psychiatric disorders. Circadian variation has been shown to affect multiple aspects of cognitive function especially executive function related to prefrontal cortex. The effects of diurnal variation in saccadic task performance and the dissociation of these effects from repetition or practice effects has not been adequately addressed. In the current study thirty healthy adults performed several saccadic eye movement tasks including visually guided saccades, antisaccades and countermanding saccades in three consecutive sessions. Participants were divided into three groups, with a different starting time of the sequence of the three sessions across groups (morning or afternoon or evening) to examine the effect of diurnal variation (time of day that the tasks were performed) separated from the effect of session repetition (practice effect). The results showed no effect of diurnal variation for all indexes of saccadic eye movement performance including accuracy (antisaccade and countermanding saccade tasks) speed (mean latency in all tasks) and stability (intra-subject standard deviation of latency in all tasks). In contrast, saccadic task repetition significantly improved accuracy, speed and stability of performance indicating the presence of practice effects in these tasks. Finally, linear mixed model analysis confirmed no interaction between diurnal variation and practice effects for all indexes of saccadic eye movement performance. In conclusion our study provides confirmation that saccadic task performance is not affected by diurnal variation related to circadian rhythms. In contrast, short term repetition of these tasks results in significant practice effects.

## Introduction

Eye movements, and in particular saccadic movements, have long been used to study a broad range of cognitive functions in experimental animals and humans. The study of saccadic movements has contributed considerably to basic neuropsychological research on cognition (Hutton 2008) as well as clinical research in neurological disorders (Müri et al. 2019; Antoniades and Spering 2024), psychiatric disorders (Bittencourt et al. 2013; Smyrnis et al. 2019) and developmental disorders (Rommelse et al. 2008; Klein et al. 2019). In recent years the study of eye movements has expanded in a broad spectrum of diverse fields such as marketing (Rothensee and Reiter 2019; Motoki et al. 2021) economics (Fiedler et al. 2019; Borozan et al. 2022) and vehicle control (Rosner et al. 2019; Groner and Kasneci 2021). Saccadic eye movement tasks provide a powerful tool for the study of cognition in all these fields because they are easy to implement (simple instructions), the properties of saccades are well described, and their neurobiological substrate is largely defined (Leigh and Kennard 2004; Leigh and Zee 2015). Furthermore, saccadic eye movements are easily reproducible, stereotyped, and automated, providing a large amount of data in a short time, in a non-invasive manner and without significant fatigue (Antoniades et al. 2013).

**Fig. 1:**
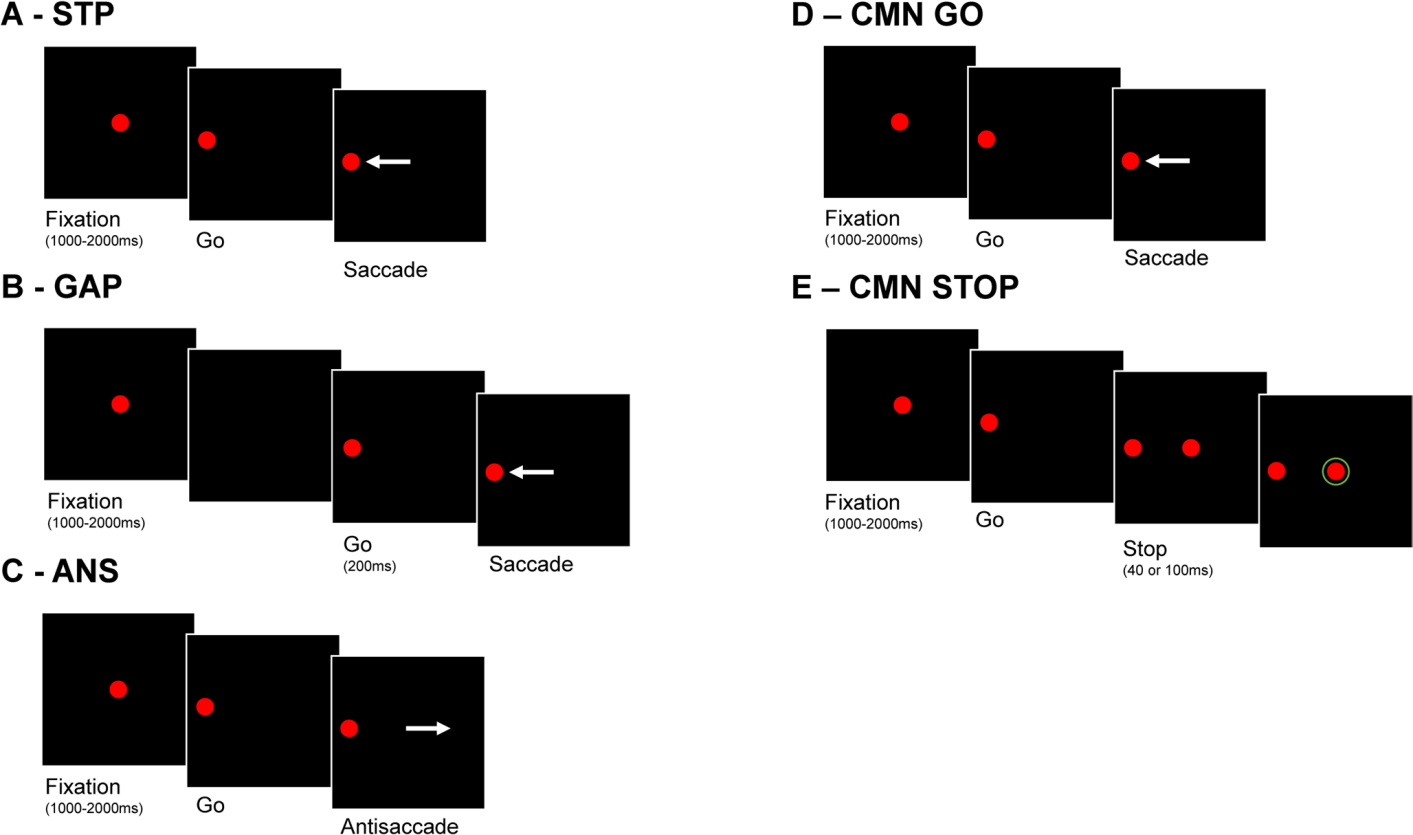
This figure presents the sequence of events in each of the 4 saccade tasks used in the current study. **A**: Step saccade task (STP); in this task the central fixation remains for 1-sec and then the peripheral target appears simultaneously with the disappearance of central fixation (target jumps) and the participant saccades to the peripheral target. **B**: Gap saccade task (GAP); same as the STP task but the central fixation target disappears 200ms before the appearance of the peripheral target. **C**: antisaccade task (ANT); same as the STP task but the participant is instructed to saccade at the mirror location from that of the peripheral target. **D**: Go trial of the countermanding (CMN) saccade task; same as the STP task (50% of trials). **E**: Stop trial of the CMN saccade task; after 40ms or 100ms from the disappearance of the central target and the simultaneous appearance of the peripheral target the central target reappears signaling the participant to stop for executing the saccade to the peripheral target (50% of trials)

The simplest saccadic eye movement task is the visually guided saccade task (step task STP, in Fig. 1). Since it would not be helpful for every visual target to elicit a saccadic movement towards it (particularly in natural environments where many stimuli exist simultaneously), time is required between sensory and motor processing to decide whether such a response is desirable, and this is reflected in the latency for each saccade (Carpenter 2004). The mean latency of the saccades measures the speed of processing in this task while the standard deviation of the latency measures the intra-subject variability of performance which is a measure of cognitive stability (Saville et al. 2011). In the visually guided saccade task with a gap (GAP, Fig. 1) the central target disappears before the onset of the peripheral target (see Fig. 1), resulting in the disengagement of attention from the central visual stimulus before planning the saccade (Saslow 1967). The prior disengagement of attention results in the “gap effect”, which is a reduction of the mean latency in this task compared to the step saccade task (Jin and Reeves 2009; Fischer and Weber 2010; Van der Stigchel et al. 2017).

In the antisaccade task (ANS Fig. 1), the subject is asked to inhibit the saccade to a visual stimulus and instead make a saccadic movement at a mirror location in the opposite direction (Hallett 1978). The percentage of directional errors is an index of performance accuracy in the task. Performance speed is measured by the mean latency of correct antisaccades, and intra-subject variability of performance is indexed by the standard deviation of the latency for correct antisaccades. The antisaccade task has been widely used as a probe to study cognitive control and executive function (Hutton and Ettinger 2006). Correct antisaccade execution requires the inhibition of the visually guided saccade, the planning of a saccade in the opposite direction (requiring a reversal of the spatial location of the saccade target) and finally the execution of the planned saccade towards the new location held in working memory (Kane et al. 2001; Unsworth et al. 2004).

Another saccadic eye movement task that engages inhibitory cognitive control is the countermanding saccade task (CMN see Fig. 1) (Hanes and Carpenter 1999). The ability to suppress the saccade in stop signal trials depends on the time between the appearance of the peripheral target and the appearance of the inhibition signal (“stop signal delay effects”). The longer the period between the two signals, the more difficult the inhibition becomes (Hanes and Carpenter 1999). The percentage of erroneous saccades in the stop signal trials indexes performance accuracy while performance speed and intra-subject variability of performance are indexed by the mean latency and standard deviation of latency respectively, in go trials. The CMN task engages voluntary movement control and inhibition (Logan and Irwin 2000; Wong-Lin et al. 2010; Salinas and Stanford 2013).

The application of these saccadic eye movement tasks to study cognition in different populations needs to address the issue of diurnal variation related to the circadian rhythm. If performance in these tasks is significantly modulated by diurnal variation, then study design should control the time of the day for data acquisition for all participants. In general, it has been shown that cognitive performance depends on the interaction of two mechanisms: an internal circadian clock and a homeostatic process. The internal clock causes fluctuations in performance that follow a period of approximately 24 h and that coincide with the variation in body temperature and cortisol secretion, while the homeostatic mechanism causes a gradual decline in performance with the time spent awake (Waterhouse 2010; Valdez et al. 2012). The circadian rhythm of cortisol secretion itself is made up from changes in the pulse amplitude of an underlying ultradian rhythm oscillating with a period of 60 to 90 min (Spencer et al. 2018). The influence of the circadian rhythm and the homeostatic mechanism has been observed in cognitive functions such as attention and working memory, each of which exhibits different patterns of fluctuation throughout the day (Valdez 2019). In addition, other studies have investigated the circadian rhythm in executive functions. The results suggest that inhibition, as measured by Go/No-go tasks, changes with the time of day. Further studies using the Stroop test concluded that inhibition and cognitive flexibility follow a circadian pattern, with the lowest performance observed early in the morning (García et al. 2012; Valdez 2019). In contrast, other studies either do not confirm the existence of diurnal variation in inhibition and cognitive flexibility (Sagaspe et al. 2006; Harrison et al. 2007) or show a different pattern of variation throughout the day (Manly et al. 2002). In overall terms, there is no consensus in the literature for the presence of diurnal variation effects on executive function. These discrepancies could arise from the fact that a broad range of processes are covered by the term ‘executive functions’. Some of these processes could be affected by diurnal variation while others would not. Some investigators suggest that diurnal variation effects are observed when greater control is required, while more automated and stereotyped processes remain unaffected (Schmidt et al. 2007).

Is there an effect of diurnal variation on the performance measures of saccadic eye movement tasks? Only a couple of studies have specifically addressed the issue of diurnal variation in saccadic eye movement task performance. Roy-Byrne et al. (1995) examined performance on ANS, STP and other oculomotor tasks at two different times of the day in 8 participants and reported no significant difference between morning and afternoon performance. In contrast, Collet et al. (2020), aiming to investigate the effects of circadian rhythm and sleep deprivation on attentional control, used the ANS and STP tasks in 10 consecutive measurements over 40 h without sleep in 21 participants. Their results supported, among other conclusions, the effect of circadian rhythm on inhibition, as they found a decrease in the percentage of incorrect responses in the ANS task over the course of a biological day. However, there was no effect on antisaccade or prosaccade latencies. A significant confounding factor in these studies where repeated measurements of saccadic tasks are necessary is the effect of repetition on performance. While performance on oculomotor tasks is generally considered to be stable over time, specific parameters of some tasks appear to change after repeated measurements. Specifically, the error rate in the ANS task decreases with repetition, either within the same session or after one week (Green et al. 2000; Smyrnis et al. 2002; Dyckman and McDowell 2005). Similarly, response time (RT) in the ANS task decreases with repetition, as does the response time of prosaccades in healthy adults trained in the task (Fischer and Weber 1992; Dyckman and McDowell 2005). Gais et al. (2008), who investigated antisaccade and prosaccade reaction times at three different times after training, showed a reduction in reaction time only 12 h after training and a maintenance of the improved performance later, without any change during training. Conversely, other studies investigating implicit learning of a sequence of saccadic movements after practice showed a reduction in response time during practice and 24 h later (Albouy et al. 2006; Meital et al. 2013). In contrast, Ettinger et al. (2003) observed no change in either the error rate in the ANS task or the response time of antisaccades and prosaccades during a training session. However, when the tasks were repeated two months later, there was a significant reduction in the error rate and an improvement in the spatial accuracy of antisaccades, with no change in the response time of antisaccades and prosaccades. Therefore, there seem to be effects of task repetition on performance of these tasks although there is discrepancy in the literature as to which tasks, which parameters, and the time frame of the emergence of these effects.

In the current study we addressed the issue of diurnal variation in the performance of four saccadic eye movement tasks ranging from simple tasks (STP and GAP, Fig. 1) to more demanding tasks (ANS, CMN see Fig. 1) that engage inhibitory cognitive control. Importantly, our study also attempts to control for the potentially confounding effect of learning across repeated test sessions that may have influenced previous work (Collet et al. 2020). Thirty healthy adults underwent three consecutive measurements of these tasks at three different time points of the day. Participants were divided into three groups varying starting time of the sequence of three measurements across groups (morning or afternoon or evening) thus dissociating diurnal variation from repetition effects. Our hypothesis was that diurnal variation would have a small or no effect on mean latency and intra-subject variability of latency for simple saccadic eye movement tasks (STP and GAP) while there would be a significant effect on these variables as well as accuracy in the performance of more demanding tasks involving inhibitory cognitive control (ANS and CMN). We also hypothesized that repetition would result in practice effects that would also be more pronounced in the more demanding tasks (ANS, CMN) compared to the simple saccade tasks (STP, GAP). We also expected these effects to be independent from the diurnal variation effects.

## Method

### Participants

Thirty healthy adults participated in the study (17 women and 13 men) aged 19–40 years (mean age: 29.8years, SD: 6.6 years). The choice for the number of participants was based on an a priori power analysis using G*power (Version 3.1.9.6). The design was a single group repeated measures ANOVA with 3 repeated measurements (corresponding to diurnal variation or repetitions). Effect size was set to 0.25 (medium), alpha probability was set to 0.05 and power was set to 0.8. Using these criteria the estimated participant number was 28, with the actual n of 30 giving a power of 0.84. Participants had an average 16.9 years of education (SD: 2.6years, range: 12–23 years). Exclusion criteria for all participants included the presence of any neurological or psychiatric disorder and the use of psychotropic or antihistamine medication. All participants were instructed to maintain their usual level of activity and to have a subjectively satisfactory level of sleep on the days on which the experiment was conducted.

The study protocol was approved by the Ethics Committee of the University Mental Health Research Institute. Participants were provided with written information about the purpose and duration of the study, the nature of the experimental tasks, and the personal data collected, which were treated anonymously and confidentially, in accordance with the legislation and the relevant provisions of the General Data Protection Regulation (GDPR) of the European Parliament and Council. Participants signed informed consent forms, informing them of the voluntary nature of their participation and their right to withdraw at any point during the study.

### Procedure

The 30 participants were divided into three groups, each consisting of ten people. The experiment for each participant consisted of three sessions over a 24-hour period during one or two calendar days, depending on the group to which the participant was assigned. In each session the participant performed all oculomotor tasks. The timing of each session was determined by the time of morning awakening based on the relevance of this schedule to internal circadian rhythms and especially the circadian variation of cortisol with highest cortisol levels at 30 min post awakening and lowest late in the evening (Debono et al. 2009). Thus, each participant underwent a morning, afternoon, and evening session at 1-, 6-, and 12-hours post-awakening, respectively. The mean absolute time for the morning measurement for all participants was 10am (SD: 1 h ), for the afternoon measurement it was 15pm (SD: 1 h ) and for the evening measurement it was 21pm (SD: 1 h). The groups, however, differed in the time of the day they started the experiment. This resulted in three groups: the first followed the sequence morning—afternoon—evening, the second followed the sequence afternoon—evening—next morning and the third followed the sequence evening—next morning—next afternoon. One member of the research group visited the participant at his/her home three times to collect the data. Weekend days or days-off from work were selected so the participant was at home. We did not use some specific instrument for quality of sleep and physical activity measurement. However, we asked the participants to try to have a good night sleep and avoid strenuous physical activity and consuming alcohol for the day of measurement, which was a weekend day during which they remained at their home for most of the time. We relied on self-reports of participants for complying with these instructions.

### Eye movement recording

Oculomotor tasks were performed using the “Saccadometer Plus” (Ober Consulting Sp. z.o.o. Poznań, Poland), a portable device, with unrestrained head movement, easily used outside of laboratory environment that detects horizontal eye movements, with sampling frequency of 1 kHz, using the technology of Direct Infra-Red Oculography. The device records infrared reflections from the medial limbus of each eye, which then are compared to produce a single measure of horizontal gaze position, under the assumption that conjugate eye movements are being made. Three visual targets are projected by the device itself by means of three laser emitting light sources located above the eyes (forehead), one centre and two peripheral ones to the right and left of the centre at equal distances of 10 degrees of visual angle. This set up produces peripheral targets at a fixed angular separation of 10 degrees from the centre target, regardless of the separation between the device and the projection surface. Measurements were performed in a quiet room under good lighting conditions with additional lighting, if needed, coming from fluorescent lights. The targets were projected on a wall at 1.5–3 m, at eye level.

### Oculomotor tasks

In each session four oculomotor tasks were performed by each participant: (a) the step visually guided saccade task (STP), (b) the gap-visually guided saccade task (GAP), (c) the antisaccade task (ANS) and (d) two blocks of the countermanding saccade task (CMN). The STP started with fixation on the central target for a period selected randomly at each trial between 1000 and 2000ms (uniformly distributed). Then the central target disappeared and simultaneously one of the peripheral targets appeared (selected randomly at each trial) and remained for a period of 1000ms. The subject was instructed to look at the peripheral target as soon as it appeared. The GAP task was identical to the STP task with the only difference being a time gap of 200ms between the disappearance of the central target and the appearance of the peripheral target. The ANS task was identical to the STP task, and the subject was instructed to look at the mirror location away from the peripheral target when this appeared. Finally, the CMN was identical to the STP task for go trials where the subject was instructed to look at the appearing peripheral target as quickly as possible, but to withhold their response if the central target reappeared (stop signal). The stop signal could appear either at 40ms after the appearance of the peripheral target or 100ms after the appearance of the peripheral target in two separate blocks of the CMN task (CMN40, CMN100).

Whilst it is recommended that different stop signals be interleaved within a block to prevent participants predicting the stop signal and so delaying their responses accordingly (Verbruggen et al. 2019), the embedded software on the occulometer we used did not allow this. Equally, the probability of the stop signal’s appearance was 50%, which was higher than has been recommended for countermanding tasks (Verbruggen et al. 2019). Given these methodological constraints, we do not attempt to calculate an absolute stop signal reaction time for our participants. The strategy of inhibiting go responses is the hallmark of the CMN task however, and so our study directly examines the magnitude of this inhibition (latency of go responses) and how successfully this allows successful countermanding (proportions of errors) for each CMN stop signal delay.

The order of the oculomotor tasks was pre-fixed and different for each participant and for each measurement of the same individual, based on a pseudo-random order predefined by protocol to ensure that there would be no order effect. Each task was completed in blocks of 100 trials, with short breaks between tasks. Before each task, there was a short training session including 10 practice trials to ensure that participants understood the task instructions and were able to perform it correctly. A calibration procedure was performed before the start of each task, using a sequence of twenty saccadic eye movements, ten to the left and ten to the right of the central fixation stimulus. We used the built-in calibration sequence of the device, readjusting the position of the tracker if the gain was not between 50 and 150 arbitrary units per degree, as recommended by the manufacturer. The calibration procedure and the 10 practice trials preceded every task repetition.

### Data preprocessing and analysis

The oculomotor data were extracted using the “Latency Meter version 6.11.0 (Ober Consulting Sp. z.o.o. Poznań, Poland)”, which allowed us the visualization and verification of the oculomotor data.

In the STP and GAP tasks, the latency for the first saccadic eye movement after the peripheral stimulus presentation was recorded. After exclusion of latencies below 80ms we computed for each subject and each session the mean latency and the standard deviation of latency separately for the GAP and STP task. We then tested for the presence of a Gap effect in mean latency and SD of latency between the GAP and STP tasks using a paired samples t-test.

In the ANS task the percentage of correct antisaccades was measured as well as the mean latency and the standard deviation of latency for correct antisaccades. Again, we excluded latencies below 80 ms.

In the CMN task we computed separately for each block (40ms and 100ms of stop signal onset) the percentage of correct responses in the stop signal trials as well as the mean and standard deviation of the latency for the go trials. Again, we excluded latencies below 80ms. We then tested for significant stop signal delay effects (using paired sample t-tests). The effect of stop signal delay for each subject was defined as the difference between blocks (40ms and 100ms stop signal delay) for each CMN parameter (percentage of correct responses for the stop signal trials, mean latency and standard deviation of latency for the go trials). The longer the delay for the stop signal in the CMN task, the more difficult it is to inhibit the saccade response.

For each index of saccadic task performance as described above we performed a one-way repeated measures ANOVA to examine the effect of diurnal variation using time of day as the independent repeated measures factor (morning, afternoon, evening). The Greenhouse-Geisser correction was used to correct p values for violations of the sphericity assumption. We also performed a Bayesian repeated measures ANOVA to compute the Bayes factor, BF_(1,0)_ favoring the alternative hypothesis that diurnal variation has a significant effect on the specific parameter and its inverse BF_(0,1)_ favoring the null hypothesis that diurnal variation has no effect on the specific parameter. The same analyses were performed to examine the effect of repetition (first, second third) as repeated measures factor on all saccade parameters. For the ANOVA and Bayesian ANOVA analyses we used the JASP 0.19.3 software.

To test for interaction effects between diurnal variation and repetition on oculomotor function indexes we used the linear mixed effects model analysis using JAMOVI (the jamovi project 2024, version 2.6). This analysis, in contrast to the standard ANOVA analysis, allows for incomplete data sets to be used. This was the case for our data set since we could not have all combinations of repetition and diurnal variation for each subject to allow for a two factor repeated measures ANOVA (with repetition and diurnal variation as the within subject repeated measures) to be used. The linear mixed effects model analysis with random intercept was used with participant as the random intercept factor and diurnal variation and repetition as the independent fixed effect factors. We report the F values for the main effect of diurnal variation, main effect of repetition and the interaction effect.

## Results

### Verification of gap effect and stop signal delay effects

The mean latency for the GAP task (mean: 143ms, SD: 19ms) was significantly smaller compared to the mean latency for the STP task (mean: 180ms, SD: 19ms) (t_89_ = 21.3, *p* <.001) confirming a significant gap effect for mean latency. The SD of latency for the GAP task (mean: 51ms, SD: 23ms) was no different form the SD of latency for the STP task (mean: 49ms, SD: 21ms) (t_89_ = 0.95, *p* =.346) showing no gap effect for the SD of latency. Thus, the effect of diurnal variation and repetition was tested only for the difference in mean latency between GAP and STP tasks.

The percent of correct responses in the CMN task with 40ms stop signal delay (mean: 88.2 SD: 8.7) was significantly larger compared to that for the CMN task with 100ms stop signal delay (mean: 83.8 SD: 8.3) (t_89_ = 5.3, *p* <.001). The mean latency for the CMN task with 40ms stop signal delay (mean: 311ms SD: 56ms) was significantly smaller compared to that for the CMN task with 100ms stop signal delay (mean: 325ms, SD: 49ms) (t_89_ = 2.87, *p* =.005). Finally, the SD of latency for the CMN task with 40ms stop signal delay (mean: 110ms SD: 34ms) was also significantly smaller compared to that for the CMN task with 100ms stop signal delay (mean: 125ms, SD: 33ms) (t_89_ = 4.99, *p* <.001). The effects of diurnal variation and repetition were tested for the difference in each one of these parameters between the two CMN tasks (effects of stop signal delay).

### Effects of diurnal variation on saccade performance parameters

#### Step and gap saccade tasks

**Fig. 2:**
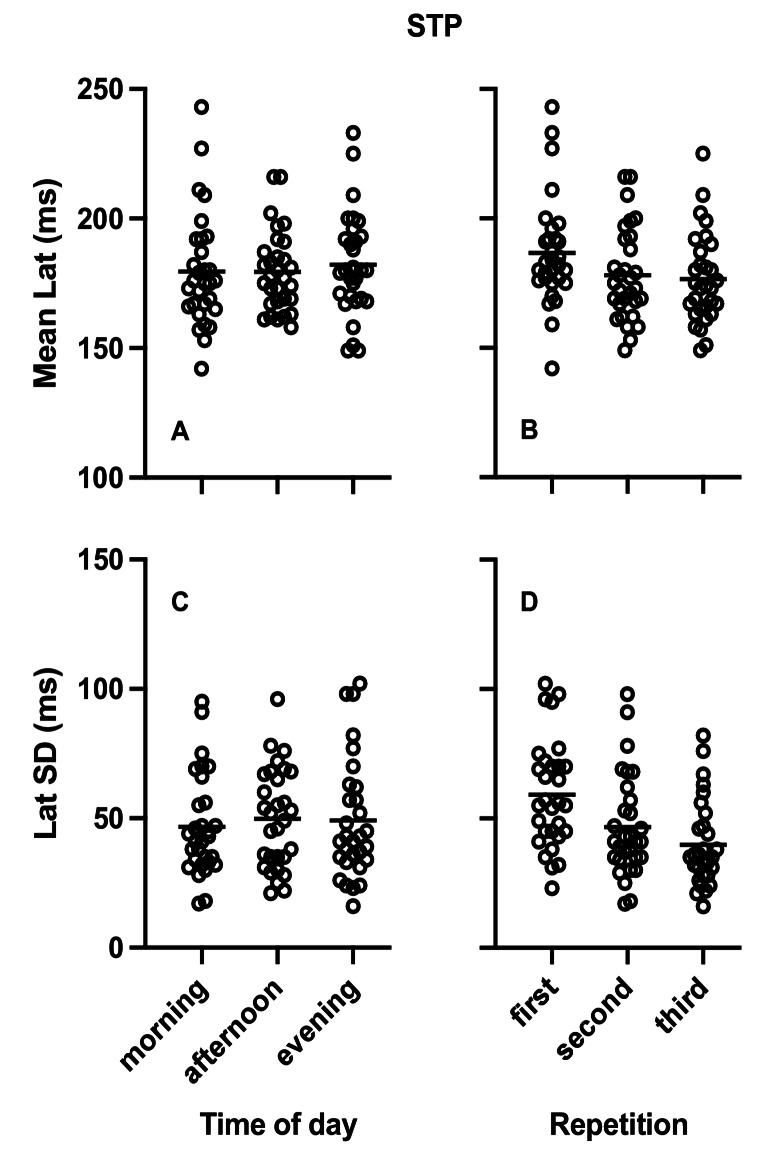
Plots of single participant data (empty circles) for Mean Latency (**A**, **B**) and Standard Deviation (SD) of latency (**C**, **D**) in the step (STP) saccade task. **A** and **C** present the data arranged by time of day while **B** and **D** present the same data arranged by repetition. Horizontal lines represent the corresponding group means

**Fig. 3:**
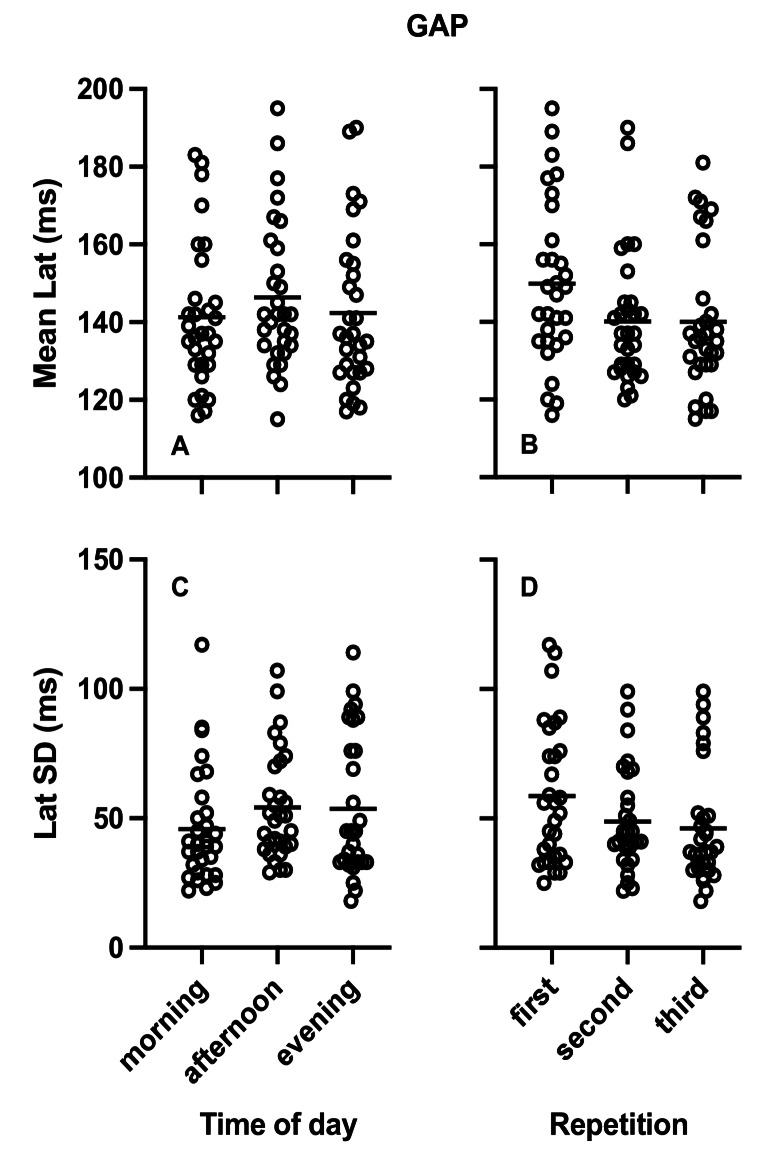
Plots of single participant data (empty circles) for Mean Latency (**A**, **B**) and Standard Deviation (SD) of latency (**C**, **D**) in the GAP saccade task. **A** and **C** present the data arranged by time of day while **B** and **D** present the same data arranged by repetition. Horizontal lines represent the corresponding group means

Table 1 presents the results of the repeated measures ANOVA analysis the effects of diurnal variation on mean latency and SD of latency for the STP and GAP task as well as the gap effect for mean latency. No diurnal variation differences were observed (Figs. 2A and C and 3A and C). The Bayesian repeated measures ANOVA also showed medium evidence favoring the null hypothesis that there was no effect of diurnal variation except for gap effect for mean latency where evidence for the null hypothesis was anecdotal.

**Table 1:** Effects of diurnal variation on saccadic task parameters

	Morning mean (SD)	Afternoon mean (SD)	Evening mean (SD)	F_2,58_ (*p*)	BF_(0,1)_*
*STP*					
Mean Lat (ms)	180 (22)	179 (15)	182 (20)	0.54 (0.582)	6.3
Lat SD (ms)	47 (20)	49 (19)	49 (23)	0.11 (0.886)	9.2
*GAP*					
Mean Lat (ms)	143 (19)	148(20)	143 (20)	1.54 (0.224)	3.1
Lat SD (ms)	47 (23)	55 (21)	55 (27)	1.69 (0.196)	2.6
*Gap effect*					
Mean Lat (ms)	37 (20)	31 (17)	39 (14)	2.72 (0.076)	1.2
*ANS*					
Correct (%)	88.5(10)	86.2(12.7)	86.2(13.5)	0.86 (0.423)	5.1
Mean Lat (ms)	272 (39)	273 (28)	273 (45)	0.02 (0.963)	10
Lat SD (ms)	67 (34)	76 (31)	69 (33)	1.22 (0.298)	3.8
*CMN40*					
Correct (%)	90.1(8.3)	86.3(9.2)	88.2(8.5)	2.51 (0.09)	1.4
Mean Lat (ms)	308 (51)	307 (52)	317 (65)	0.75 (0.475)	5.6
Lat SD (ms)	105 (30)	110 (37)	115 (34)	0.98 (0.38)	4.5
*CMN100*					
Correct (%)	83.7 (8.5)	82.7 (8.9)	85.1 (7.4)	1.04 (0.351)	3.9
Mean Lat (ms)	320 (47)	328 (48)	327 (52)	0.42 (0.651)	7.2
Lat SD (ms)	122 (33)	127 (33)	126 (36)	0.27 (0.761)	8
*Stop signal delay effect*					
Correct (%)	6.4 (8.3)	3.6 (7.9)	3.2 (7.1)	1.93 (0.156)	2.1
Mean Lat (ms)	11 (45)	21 (45)	9.4 (49)	0.48 (0.597)	6.4
Lat SD (ms)	17 (28)	17 (33)	11 (27)	0.42 (0.655)	6.9

*The Bayesian Factor in favor of the null hypothesis (0,1) is presented. BF > 1 and BF < = 3 indicates anecdotal evidence, BF > 3 and < = 10 indicates medium evidence, BF > 10 and BF < = 30 indicates strong evidence, BF > 30 and < = 100 indicates very strong evidence and BF > 100 indicates extremely strong evidence (Kelter 2020)

#### Antisaccade task

**Fig. 4:**
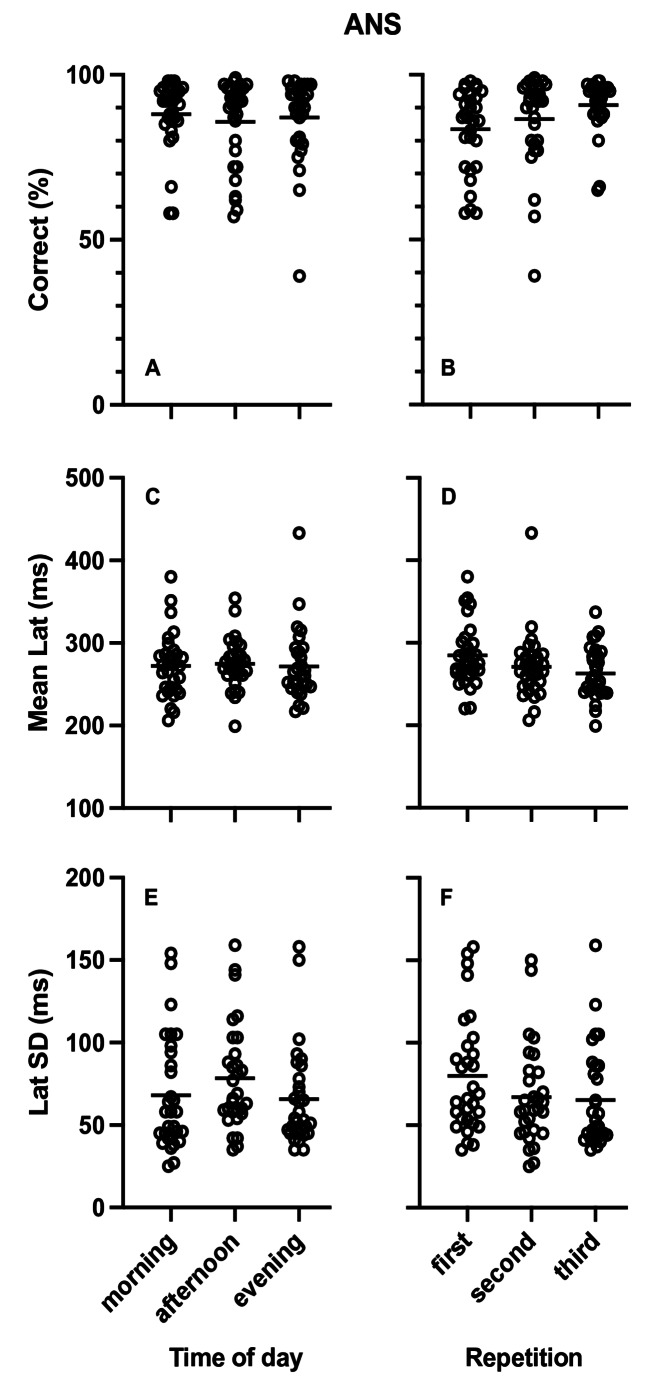
Plots of single participant data (empty circles) for percent of correct responses (**A**, **B**) Mean Latency (**C**, **D**) and Standard Deviation (SD) of latency (**E**, **F**) in the antisaccade (ANT) task. **A**, **C** and **E** present the data arranged by time of day while **B**, **D** and **F** present the same data arranged by repetition. Horizontal lines represent the corresponding group means

Table 1 presents the results of the repeated measures ANOVA analysis for the effects of diurnal variation on percent of correct responses, mean latency and SD of latency for the ANS task. No diurnal variation differences were observed (Fig. 4A, C and E). The Bayesian repeated measures ANOVA also showed medium evidence favoring the null hypothesis that there was no effect of diurnal variation.

#### Countermanding saccade tasks

**Fig. 5:**
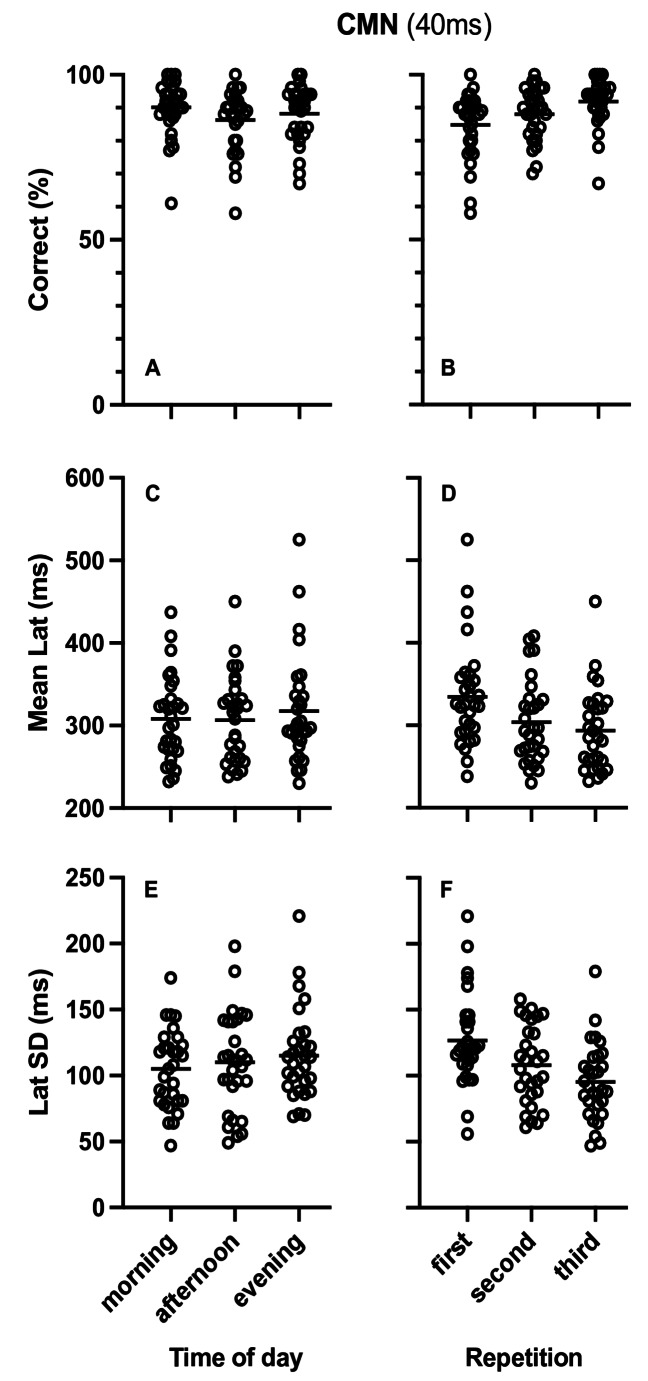
Plots of single participant data (empty circles) for percent of correct responses (**A**, **B**) Mean Latency (**C**, **D**) and Standard Deviation (SD) of latency (**E**, **F**) in the countermanding saccade task with 40ms stop signal delay (CMN 40ms). **A**, **C** and **E** present the data arranged by time of day while **B**, **D** and **F** present the same data arranged by repetition. Horizontal lines represent the corresponding group means

**Fig. 6:**
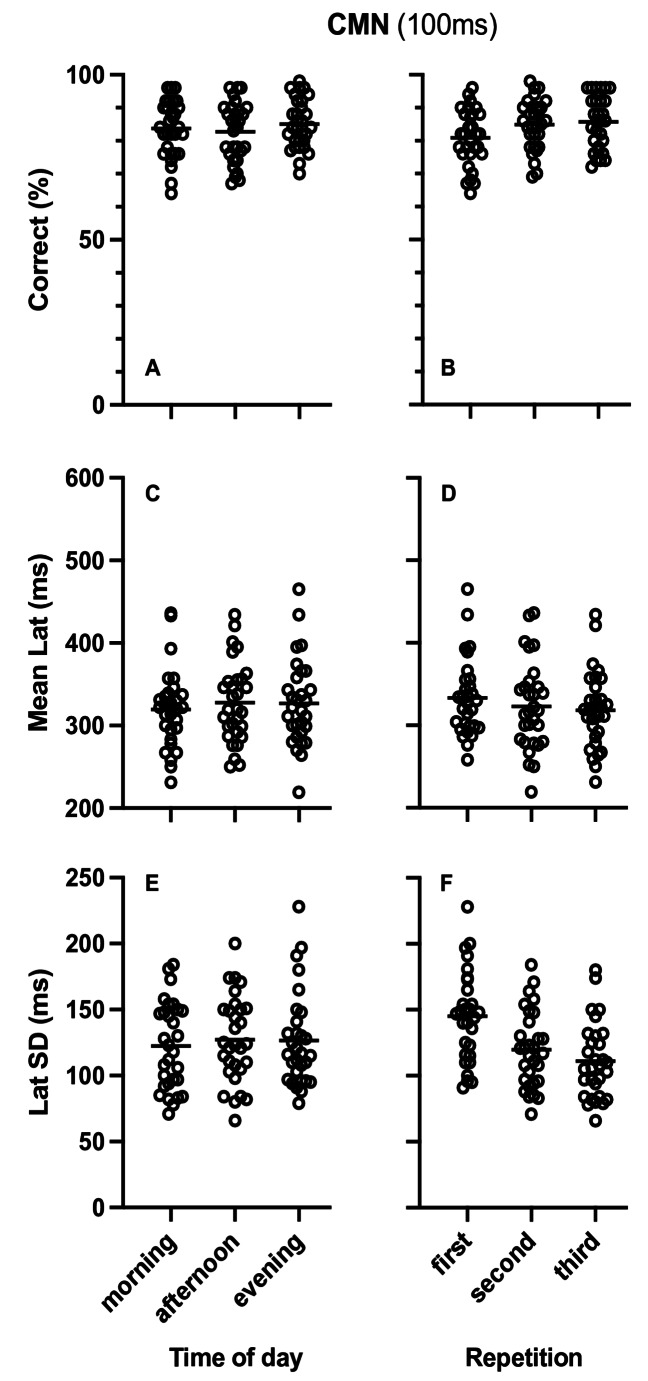
Plots of single participant data (empty circles) for percent of correct responses (**A**, **B**) Mean Latency (**C**, **D**) and Standard Deviation (SD) of latency (**E**, **F**) in the countermanding saccade task with 100ms stop signal delay (CMN 100ms). **A**, **C** and **E** present the data arranged by time of day while** B**,** D** and** F** present the same data arranged by repetition. Horizontal lines represent the corresponding group means

Table 1 presents the results of the repeated measures ANOVA analysis for the effects of diurnal variation on percent of correct responses, mean latency and SD of latency for the CMN tasks with 40ms and 100ms stop signal delay, as well as the effects of stop signal delay on percent correct responses, mean latency and SD of latency. As can be seen in Table 1 there were no significant effects of diurnal variation (Fig. 5A, C and E for CMN40 and Fig. 6A, C and E for CMN100). The Bayesian repeated measures ANOVA also showed medium evidence favoring the null hypothesis except for the percent of correct responses in the CMN task with 40ms stop signal delay and the effect of stop signal delay on percent of correct responses where evidence for the null hypothesis was anecdotal.

### Effects of repetition on saccade performance parameters

#### Step and gap saccade tasks

Table 2 presents the results of the repeated measures ANOVA for the effects of repetition on mean latency and SD of latency in the STP and GAP tasks. Repetition resulted in significant decrease of mean latency and SD of latency in both saccade tasks (see also Figs. 2B and D and 3B and D). The Bayesian repeated measures ANOVA showed evidence favoring the alternative hypothesis confirming significant effects and ranging from strong to extremely strong except for the SD of latency in the GAP task where evidence was anecdotal. There was no significant effect of repetition on the gap effect of mean latency and the Bayesian analysis showed medium evidence favoring the null hypothesis.

**Table 2:** Effects of repetition on saccadic task parameters

	First mean (SD)	Second mean (SD)	Third mean (SD)	F_2,58_ (*p*)	BF_(1,0)_*
*STP*					
Mean Lat (ms)	187 (21)	178 (18)	176 (17)	9 (< 0.001)	72.6
Lat SD (ms)	59 (21)	47 (20)	40 (16)	22.6 (< 0.001)	209933.4
*GAP*					
Mean Lat (ms)	149.8(20.9)	140.2(17.1)	140(18.4)	6.6 (0.005)	13.8
Lat SD (ms)	59 (27)	49 (20)	46 (22)	3.9 (0.029)	2.1
*Gap effect*					
Mean Lat (ms)	37 (17)	38 (19)	36 (12)	0.09 (0.893)	0.11**
*ANS*					
Correct (%)	84 (13)	86 (14)	91 (8)	8.6 (< 0.001)	52.8
Mean Lat (ms)	285 (39)	271 (40)	263 (31)	8.3 (< 0.001)	42.5
Lat SD (ms)	80 (35)	67 (30)	65 (30)	3.4 (0.045)	1.4
*CMN40*					
Correct (%)	85 (10)	88 (8)	92 (7)	11.2 (< 0.001)	310.9
Mean Lat (ms)	335 (62)	304 (50)	294 (49)	14.1 (< 0.001)	1748.1
Lat SD (ms)	127 (35)	108 (30)	95 (29)	13.7 (< 0.001)	1696
*CMN100*					
Correct (%)	81 (8)	85 (8)	86 (8)	5.4 (0.007)	6.3
Mean Lat (ms)	333 (47)	323 (53)	318 (47)	1.3 (0.287)	0.269
Lat SD (ms)	145 (34)	120 (30)	111 (29)	20.6 (< 0.001)	87,249
*Stop signal delay effects*					
Correct (%)	4 (8)	3 (7)	6 (8)	1.7 (0.199)	0.391**
Mean Lat (ms)	2 (49)	19 (51)	25 (35)	2.5 (0.096)	1.1
Lat SD (ms)	18 (26)	12 (34)	16 (27)	0.4 (0.662)	0.145**

*The Bayesian Factor in favor of the alternative hypothesis (1,0) is presented. BF > 1 and BF < = 3 indicates anecdotal evidence, BF > 3 and < = 10 indicates medium evidence, BF > 10 and BF < = 30 indicates strong evidence, BF > 30 and < = 100 indicates very strong evidence and BF > 100 indicates extremely strong evidence (Kelter 2020)

**BF favors the null hypothesis (BF < 1)

#### Antisaccade task

Table 2 presents the results of the repeated measures ANOVA for the effects of repetition on percent of correct responses, mean latency and SD of latency in the ANS task. Repetition resulted in significant increase of correct responses, decrease of mean latency and decrease SD of latency in the ANS task (Fig. 4B, D and F). The Bayesian repeated measures ANOVA showed very strong evidence favoring the alternative hypothesis confirming the significant effects except for the SD of latency where evidence was anecdotal.

#### Countermanding saccade tasks

Table 2 presents the results of the repeated measures ANOVA for the effects of repetition on percent of correct responses, mean latency and SD of latency in the CMN task with 40ms and 100ms stop signal delay. Repetition resulted in significant increase of correct responses and decrease SD of latency in both CMN tasks (Fig. 5B and F for CMN 40 and Fig. 6B and F for CMN 100). Mena latency also significantly decreased with repetition in the CMN task with 40ms stop signal delay (Fig. 5D) but there was no effect of repetition on the mean latency for the CMN task with 100ms stop signal delay (Fig. 6D). The Bayesian repeated measures ANOVA showed medium and extremely strong evidence favoring the alternative hypothesis confirming the significant effects except in the case for the mean latency of saccades in the CMN task with 100ms delay where the BF favored the null hypothesis with medium size evidence. Finally there was no effect of repetition on the stop signal delay effects and the Bayesian analysis showed anecdotal evidence for the alternative hypothesis for the effect on mean latency and anecdotal evidence favoring the null hypothesis for the effect on percent of correct responses (BF_(0,1)_ = 2.5) and medium evidence favoring the null hypothesis for the effect on SD of latency (BF_(0,1)_ = 6.9).

#### Interaction between diurnal variation and repetition

Table 3 presents the results of the linear mixed effects model analysis. The analysis confirmed the non-significant results of the ANOVA analysis for the main effect of diurnal variation on saccade task parameters except for the percent of correct responses in the CMN task with 40ms delay where a significant effect was found. Of note is this single significant effect in the 17 comparisons performed is close to the number expected by chance alone (1 in 20). Post hoc comparisons further indicated that the percent of correct responses was significantly higher in the afternoon compared to morning ( t_54_ = 2.6 *p* =.034) but there was no difference between morning and evening ( t_54_ = 1.2 *p* =.61) and between afternoon and evening ( t_54_ = 1.3 *p* =.564). The analysis also confirmed all significant effects of repetition on saccade parameters and included also a significant effect of stop signal delay on the percent of correct responses where this effect increased with repetition. More importantly this analysis showed no significant interaction between diurnal variation and repetition for all saccadic task parameters for all tasks.

**Table 3:** Results of the mixed effects linear model analysis; interaction of diurnal variation and repetition effects

	Diurnal variation main effect	Repetition main effect	Interaction effect
*STP*			
Mean Lat (ms)	F_2,54_ = 0.68, *p* =.512	F_2,54_ = 8.7, *p* <.001	F_4,38_ = 0.26, *p* =.899
Lat SD (ms)	F_2,54_ = 0.6, *p* =.552	F_2,54_ = 22.3, *p* <.001	F_4,39_ = 0.49, *p* =.739
*GAP*			
Mean Lat (ms)	F_2,54_ = 1.48, *p* =.237	F_2,54_ = 6.51, *p* =.003	F_4,40_ = 0.16, *p* =.955
Lat SD (ms)	F_2,54_ = 2.02, *p* =.142	F_2,54_ = 3.97, *p* =.025	F_4,43_ = 0.33, *p* =.853
*Gap effect*			
Mean Lat (ms)	F_2,54_ = 2.03, *p* =.142	F_2,54_ = 0.01, *p* =.92	F_4,44_ = 0.38, *p* =.821
*ANS*			
Correct (%)	F_2,54_ = 0.79, *p* =.460	F_2,54_ = 8.28, *p* <.001	F_4,39_ = 0.73, *p* =.579
Mean Lat (ms)	F_2,54_ = 0.23, *p* =.793	F_2,54_ = 8.66, *p* <.001	F_4,39_ = 1.91, *p* =.128
Lat SD (ms)	F_2,54_ = 2.38, *p* =.102	F_2,54_ = 3.43, *p* =.039	F_4,42_ = 0.21, *p* =.931
*CMN40*			
Correct (%)	F_2,54_ = 3.43, *p* =.039	F_2,54_ = 12, *p* <.001	F_4,41_ = 1.39, *p* =.254
Mean Lat (ms)	F_2,54_ = 1.07, *p* =.35	F_2,54_ = 13.8, *p* <.001	F_4,39_ = 0.44, *p* =.777
Lat SD (ms)	F_2,54_ = 1.4, *p* =.256	F_2,54_ = 13.7, *p* <.001	F_4,42_ = 0.31, *p* =.87
*CMN100*			
Correct (%)	F_2,54_ = 1.24, *p* =.298	F_2,54_ = 5.61, *p* =.006	F_4,42_ = 1.93, *p* =.123
Mean Lat (ms)	F_2,54_ = 0.43, *p* =.654	F_2,54_ = 1.23, *p* =.3	F_4,42_ = 0.78, *p* =.546
Lat SD (ms)	F_2,54_ = 0.43, *p* =.65	F_2,54_ = 19.5, *p* <.001	F_4,41_ = 0.41, *p* =.796
*Stop signal delay effect*			
Correct (%)	F_2,54_ = 1.62, *p* =.208	F_2,54_ = 4.91, *p* =.011	F_4,36_ = 0.85, *p* =.504
Mean Lat (ms)	F_2,81_ = 0.54, *p* =.582	F_2,81_ = 2.68, *p* =.075	F_4,81_ = 0.5, *p* =.735
Lat SD (ms)	F_2,54_ = 0.4, *p* =.672	F_2,54_ = 0.38, *p* =.683	F_4,48_ = 0.77, *p* =.549

## Discussion

This study investigated the effects of diurnal variation in various indexes of performance in saccadic eye movement tasks including visually guided saccades (step and gap paradigms), antisaccades and countermanding saccades. To discriminate the effects of diurnal variation from the confounding effects of task repetition (practice effects) we divided the 30 study participants into three groups of 10 participants. Each group performed the experiment three times, starting at a different time of the day to ensure that all possible combinations of order (first-second-third) and time of measurement (morning-afternoon-evening) were equally represented. This design ensured the disentangling of the effect of time of day (diurnal variation), from the effect of repetition (practice effect).

The results of the repeated measures ANOVA analysis indicated that there was no diurnal variation in performance for all indexes of performance in all saccadic eye movement tasks and these results were further corroborated by the Bayesian factor analysis. There was a significant effect of diurnal variation on the percent of correct responses in the CMN task with 40ms stop signal delay that was observed only in the linear mixed model analysis, although around one significant effect would have been anticipated by chance alone given the 17 analyses performed. Furthermore, this effect was driven by a significant increase in accuracy in the afternoon session compared only to the morning session which does not follow a circadian pattern. Additionally, our analysis showed no effect of diurnal variation on the gap effect (the difference in mean latency between the step saccade and gap saccade tasks) and the stop signal delay effects in the countermanding saccade task (difference in accuracy, speed and intra-subject variability of speed between the CMN task with 40ms stop signal delay and the CMN task with 100ms stop signal delay). Finally, the linear mixed model analysis showed that there was no significant interaction of diurnal variation and practice effects for all saccadic task variables. This was an important finding that validates our study design which aimed at effectively dissociating the effects of diurnal variation from practice effects on saccadic task performance.

These results confirm our hypothesis that diurnal variation would not alter simple saccadic eye movement tasks such as the STP and GAP which rely on stereotyped automatic processing. On the other hand, the absence of diurnal variation on the more demanding antisaccade and countermanding saccade tasks as well as the effects of stop signal delay in the CMN tasks refutes our initial hypothesis that diurnal variation would affect more demanding saccade tasks that involve inhibitory cognitive control which is considered as an “executive” function.

Only two previous studies have directly investigated the effects of diurnal variation on saccadic eye movement task performance. Roy-Byrne et al. (1995) tested 8 subjects in a visually guided saccade and antisaccade task (among other oculomotor tasks including smooth eye pursuit and visual fixation tasks). In that study participants were tested four times at weekly to bimonthly intervals, twice in the morning and twice in the afternoon in random order across subjects. The authors reported no effect of diurnal variation in any of the indexes measured including mean latency for visually guided saccades and antisaccades and percentage of error saccades towards the visual target (reflexive saccades) in the antisaccade task. Our study provides a strong confirmation for these previous results showing the absence of diurnal variation effects in a much larger sample, using Bayesian statistics to confirm the negative effects and extending the results to include indexes of intra-subject performance stability (standard deviation of latency). More importantly our study design allowed us to provide direct evidence for the dissociation of diurnal variation and practice effects. Our study also extends these findings to the gap saccade task and the countermanding saccade task that were not included in the study of Roy-Byrne et al. (1995).

Another more recent study (Collet et al. 2020) used a sleep deprivation protocol where visually guided saccades and antisaccades were measured in 24 subjects within a period of 40 h of sleep deprivation in a restricted laboratory environment. The authors report that diurnal variation had a significant effect on antisaccade accuracy during this long period of continuous monitoring with accuracy increasing from morning to evening and then decreasing again in the biological night period towards the previous biological morning levels. Although the authors suggest that these effects are due to diurnal variation the continuous recording over 40 h of sleep deprivation is not relevant to the most used paradigms of administration of these tasks that are administered once during a day and of course with no sleep deprivation. In that long term repeated application of these tasks the authors report that practice effects were ruled out because the first and the fifth measurement of the tasks (corresponding to the same biological time of day) did not differ in performance but practice effects during this long-repeated administration could be present in a different time frame and could be confounded with fatigue effects presenting later.

In our study we confirmed the presence of significant practice effects with repetition of saccadic eye movement tasks that were independent from diurnal variation. Repeated administration resulted in faster speed (reduced latency) in all saccadic tasks except in the countermanding saccade task with 100ms stop signal delay. Repeated administration also resulted in increased performance stability (reduced intra-subject standard deviation of latency) in all tasks. Finally repeated administration resulted in increased accuracy in the antisaccade and countermanding saccade tasks. In contrast we found no effect of practice on the gap effect and the stop signal delay effect of the countermanding saccade task.

The presence of practice effects in saccadic eye movement tasks has been investigated in previous studies. Three previous studies reported an improvement of antisaccade accuracy reduction of errors) with repetition (Green et al. 2000; Ettinger et al. 2003; Radant et al. 2007) but no difference in performance speed (antisaccade mean latency). Dyckman and McDowell (2005) examined for practice effects following training in the visually guided saccade and antisaccade tasks and showed improvement of both speed (reduced mean latency) and accuracy (reduced errors) with practice in both tasks. These findings are consistent with the results of our study. Our study also showed similar practice effects in the countermanding saccade task which has not been previously investigated. It is important here to note that our study reports significant practice effects in a short time interval of hours compared to some previous studies that report practice effects after a long time interval (Ettinger et al. 2003). This suggests that practice effects can be present in multiple time scales ranging from within a task block to hours to months.

Of note is that we observed performance increases with practice even though all tasks at all sessions were preceded by a training run. This suggests the actions of a slower learning mechanism than that which provides initial task familiarity, similar to the between-session improvement in task performance seen in perceptual learning experiments (McKendrick and Battista 2013). It has been shown that oculomotor tasks initially requiring some conscious control can become progressively automated over time, with specific brain areas responsible for this automated task processing (Isoda and Hikosaka 2007; Hikosaka and Isoda 2008). As such, initial participant instructions and training runs may have been sufficient for achieving conscious control to perform the task correctly but presumably were insufficient for full task automation. Automation is not oculomotor per se, however, as performance benefits from automated actions learned in non-oculomotor domains can be rapidly transferred to the oculomotor domain (Stainer et al. 2016).

In conclusion, the current study provided strong evidence for the absence of diurnal variation effects in the performance of saccadic eye movement tasks that are commonly used in cognition research in several fields. This finding is important in two ways. First it provides evidence that circadian rhythms that have been suggested to influence cognitive performance and especially the performance of executive tasks related to prefrontal cortical function do not seem to influence saccadic eye movement tasks even those that involve executive prefrontal function such as the antisaccade and the countermanding saccade tasks. Second it provides evidence that that diurnal variation is not a confounding factor in the design of studies that use saccadic eye movement tasks for measuring cognitive function. Finally, our study confirmed the presence of significant practice effects in the performance of saccadic eye movement tasks that should be considered as a potential confounding factor in the design of studies using these tasks for assessing cognitive performance over time.

## Data Availability

The data that support the findings of this study are available from the corresponding author upon reasonable request.

## References

[CR1] Albouy G, Ruby P, Phillips C, Luxen A, Peigneux P, Maquet P (2006) Implicit oculomotor sequence learning in humans: time course of offline processing. Brain Res 1090:163–171. 10.1016/j.brainres.2006.03.076 16677617 10.1016/j.brainres.2006.03.076

[CR3] Antoniades CA, Spering M (2024) Eye movements in parkinson’s disease: from neurophysiological mechanisms to diagnostic tools. Trends Neurosci 47:71–83. 10.1016/j.tins.2023.11.001 38042680 10.1016/j.tins.2023.11.001

[CR2] Antoniades C, Ettinger U, Gaymard B et al (2013) An internationally standardised antisaccade protocol. Vis Res 84:1–5. 10.1016/j.visres.2013.02.007 23474300 10.1016/j.visres.2013.02.007

[CR4] Bittencourt J, Velasques B, Teixeira S et al (2013) Saccadic eye movement applications for psychiatric disorders. Neuropsychiatr Dis Treat 9:1393–1409. 10.2147/ndt.S45931 24072973 10.2147/NDT.S45931 PMC3783508

[CR5] Borozan M, Loreta C, Riccardo P (2022) Eye-tracking for the study of financial decision-making: A systematic review of the literature. J Behav Exper Finance 35:100702. 10.1016/j.jbef.2022.100702

[CR6] Carpenter RH (2004) Contrast, probability, and saccadic latency; evidence for independence of detection and decision. Curr Biol 14:1576–1580. 10.1016/j.cub.2004.08.058 15341745 10.1016/j.cub.2004.08.058

[CR7] Collet J, Ftouni S, Clough M, Cain SW, Fielding J, Anderson C (2020) Differential impact of sleep deprivation and circadian timing on reflexive versus inhibitory control of attention. Sci Rep 10:7270. 10.1038/s41598-020-63144-y 32350303 10.1038/s41598-020-63144-y PMC7190648

[CR8] Debono M, Ghobadi C, Rostami-Hodjegan A et al (2009) Modified-release hydrocortisone to provide circadian cortisol profiles. J Clin Endocrinol Metab 94:1548–1554. 10.1210/jc.2008-2380 19223520 10.1210/jc.2008-2380 PMC2684472

[CR9] Dyckman KA, McDowell JE (2005) Behavioral plasticity of antisaccade performance following daily practice. Exp Brain Res 162:63–69. 10.1007/s00221-004-2105-9 15551081 10.1007/s00221-004-2105-9

[CR10] Ettinger U, Kumari V, Crawford TJ, Davis RE, Sharma T, Corr PJ (2003) Reliability of smooth pursuit, fixation, and saccadic eye movements. Psychophysiology 40:620–628. 10.1111/1469-8986.00063 14570169 10.1111/1469-8986.00063

[CR11] Fiedler S, Ettinger U, Weber B (2019) Neuroeconomics. In: Klein C, Ettinger U (eds) Eye movement research. Springer, Cham, pp 857–882

[CR12] Fischer B, Weber H (1992) Characteristics of anti saccades in man. Exp Brain Res 89:415–424. 10.1007/BF00228257 1623983 10.1007/BF00228257

[CR13] Fischer B, Weber H (2010) Express saccades and visual attention. Behav Brain Sci 16:553–567. 10.1017/s0140525x00031575

[CR14] Gais S, Koster S, Sprenger A, Bethke J, Heide W, Kimmig H (2008) Sleep is required for improving reaction times after training on a procedural visuo-motor task. Neurobiol Learn Mem 90:610–615. 10.1016/j.nlm.2008.07.016 18723102 10.1016/j.nlm.2008.07.016

[CR15] García A, Ramírez C, Martínez B, Valdez P (2012) Circadian rhythms in two components of executive functions: cognitive Inhibition and flexibility. Biol Rhythm Res 43:49–63. 10.1080/09291016.2011.638137

[CR16] Green JF, King DJ, Trimble KM (2000) Antisaccade and smooth pursuit eye movements in healthy subjects receiving Sertraline and lorazepam. J Psychopharmacol 14:30–36. 10.1177/026988110001400103 10757250 10.1177/026988110001400103

[CR17] Groner R, Kasneci E (2021) Eye movements in real and simulated driving and navigation control - Foreword to the special issue. J Eye Mov Res 12. 10.16910/jemr.12.3.0 10.16910/jemr.12.3.0 PMC8182438 34122742

[CR18] Hallett PE (1978) Primary and secondary saccades to goals defined by instructions. Vis Res 18:1279–1296. 10.1016/0042-6989(78)90218-3 726270 10.1016/0042-6989(78)90218-3

[CR19] Hanes DP, Carpenter RH (1999) Countermanding saccades in humans. Vis Res 39:2777–2791. 10.1016/s0042-6989(99)00011-5 10492837 10.1016/s0042-6989(99)00011-5

[CR20] Harrison Y, Jones K, Waterhouse J (2007) The influence of time awake and circadian rhythm upon performance on a frontal lobe task. Neuropsychologia 45:1966–1972. 10.1016/j.neuropsychologia.2006.12.012 17275040 10.1016/j.neuropsychologia.2006.12.012

[CR21] Hikosaka O, Isoda M (2008) Brain mechanisms for switching from automatic to controlled eye movements. Prog Brain Res 171:375–382. 10.1016/s0079-6123(08)00655-9 18718329 10.1016/S0079-6123(08)00655-9 PMC2747307

[CR22] Hutton SB (2008) Cognitive control of saccadic eye movements. Brain Cogn 68:327–340. 10.1016/j.bandc.2008.08.021 19028265 10.1016/j.bandc.2008.08.021

[CR23] Hutton SB, Ettinger U (2006) The antisaccade task as a research tool in psychopathology: a critical review. Psychophysiology 43:302–313. 10.1111/j.1469-8986.2006.00403.x 16805870 10.1111/j.1469-8986.2006.00403.x

[CR24] Isoda M, Hikosaka O (2007) Switching from automatic to controlled action by monkey medial frontal cortex. Nat Neurosci 10:240–248. 10.1038/nn1830 17237780 10.1038/nn1830

[CR25] Jin Z, Reeves A (2009) Attentional release in the saccadic gap effect. Vis Res 49:2045–2055. 10.1016/j.visres.2009.02.015 19268494 10.1016/j.visres.2009.02.015

[CR26] Kane MJ, Bleckley MK, Conway AR, Engle RW (2001) A controlled-attention view of working-memory capacity. J Exp Psychol Gen 130:169–183. 10.1037/0096-3445.130.2.169 11409097 10.1037//0096-3445.130.2.169

[CR57] Kelter R (2020) Bayesian alternatives to null hypothesis significance trsting in biomedical research: a non-technical ntroduction to Bayesian inference with JASP. BMC Med Res Methodol 20:142. 10.1186/s12874-020-00980-6 10.1186/s12874-020-00980-6 PMC7275319 32503439

[CR27] Klein C, Seernani D, Ioannou C, Schulz-Zhecheva Y, Biscaldi M, Kavšek M (2019) Typical and atypical development of eye movements. In: Klein C, Ettinger U (eds) Eye movement research. Springer, Cham, pp 635–701

[CR28] Leigh RJ, Kennard C (2004) Using saccades as a research tool in the clinical neurosciences. Brain 127:460–477. 10.1093/brain/awh035 14607787 10.1093/brain/awh035

[CR29] Leigh RJ, Zee DS (2015) The neurology of eye movements. Oxford University Press

[CR30] Logan GD, Irwin DE (2000) Don’t look! Don’t touch! Inhibitory control of eye and hand movements. Psychon Bull Rev 7:107–112. 10.3758/BF03210728 10780023 10.3758/bf03210728

[CR32] Manly T, Lewis GH, Robertson IH, Watson PC, Datta A (2002) Coffee in the cornflakes: time-of-day as a modulator of executive response control. Neuropsychologia 40:1–6. 10.1016/s0028-3932(01)00086-0 11595257 10.1016/s0028-3932(01)00086-0

[CR33] McKendrick AM, Battista J (2013) Perceptual learning of contour integration is not compromised in the elderly. J Vis 13:5. 10.1167/13.1.5 23291645 10.1167/13.1.5

[CR34] Meital N, Korinth SP, Karni A (2013) Plasticity in the adult oculomotor system: offline consolidation phase gains in saccade sequence learning. Brain Res 1528:42–48. 10.1016/j.brainres.2013.07.013 23867864 10.1016/j.brainres.2013.07.013

[CR35] Motoki K, Saito T, Onuma T (2021) Eye-tracking research on sensory and consumer science: A review, pitfalls and future directions. Food Res Int 145:110389. 10.1016/j.foodres.2021.110389 34112392 10.1016/j.foodres.2021.110389

[CR31] Müri R, Cazzoli D, Nyffeler T (2019) Eye movements in neurology. In: Klein C, Ettinger U (eds) Eye movement research. Springer, Cham, pp 749–774

[CR36] Radant AD, Dobie DJ, Calkins ME et al (2007) Successful multi-site measurement of antisaccade performance deficits in schizophrenia. Schizophr Res 89:320–329. 10.1016/j.schres.2006.08.010 17023145 10.1016/j.schres.2006.08.010

[CR37] Rommelse NN, Van der Stigchel S, Sergeant JA (2008) A review on eye movement studies in childhood and adolescent psychiatry. Brain Cogn 68:391–414. 10.1016/j.bandc.2008.08.025 18835079 10.1016/j.bandc.2008.08.025

[CR38] Rosner A, Franke T, Platten F, Attig C (2019) Eye movements in vehicle control. In: Klein C, Ettinger U (eds) Eye movement research. Springer, Cham, pp 929–969

[CR39] Rothensee M, Reiter P (2019) Neuromarketing. In: Klein C, Ettinger U (eds) Eye movement research. Springer, Cham, pp 819–855

[CR40] Roy-Byrne P, Radant A, Wingerson D, Cowley DS (1995) Human oculomotor function: reliability and diurnal variation. Biol Psychiatry 38:92–97. 10.1016/0006-3223(94)00225-R 7578655 10.1016/0006-3223(94)00225-R

[CR41] Sagaspe P, Sanchez-Ortuno M, Charles A, Taillard J, Valtat C, Bioulac B, Philip P (2006) Effects of sleep deprivation on Color-Word, emotional, and specific Stroop interference and on self-reported anxiety. Brain Cogn 60:76–87. 10.1016/j.bandc.2005.10.001 16314019 10.1016/j.bandc.2005.10.001

[CR42] Salinas E, Stanford TR (2013) The countermanding task revisited: fast stimulus detection is a key determinant of psychophysical performance. J Neurosci 33:5668–5685. 10.1523/JNEUROSCI.3977-12.2013 23536081 10.1523/JNEUROSCI.3977-12.2013 PMC3650622

[CR43] Saslow MG (1967) Effects of components of displacement-step stimuli upon latency for saccadic eye movement. J Opt Soc Am 57:1024–1029. 10.1364/josa.57.001024 6035296 10.1364/josa.57.001024

[CR44] Saville CW, Dean RO, Daley D, Intriligator J, Boehm S, Feige B, Klein C (2011) Electrocortical correlates of intra-subject variability in reaction times: average and single-trial analyses. Biol Psychol 87:74–83. 10.1016/j.biopsycho.2011.02.005 21335053 10.1016/j.biopsycho.2011.02.005

[CR45] Schmidt C, Collette F, Cajochen C, Peigneux P (2007) A time to think: circadian rhythms in human cognition. Cogn Neuropsychol 24:755–789. 10.1080/02643290701754158 18066734 10.1080/02643290701754158

[CR47] Smyrnis N, Evdokimidis I, Stefanis NC et al (2002) The antisaccade task in a sample of 2,006 young males. II. Effects of task parameters. Exp Brain Res 147:53–63. 10.1007/s00221-002-1207-5 12373369 10.1007/s00221-002-1207-5

[CR46] Smyrnis N, Amado I, Krebs M-O, Sweeney JA (2019) Eye movements in psychiatry. In: Klein C, Ettinger U (eds) Eye movement research. Springer, Cham, pp 703–748

[CR48] Spencer RL, Chun LE, Hartsock MJ, Woodruff ER (2018) Glucocorticoid hormones are both a major circadian signal and major stress signal: how this shared signal contributes to a dynamic relationship between the circadian and stress systems. Front Neuroendocr 49:52–71. 10.1016/j.yfrne.2017.12.005 10.1016/j.yfrne.2017.12.005 29288075

[CR49] Stainer MJ, Carpenter RHS, Brotchie P, Anderson AJ (2016) Sequences show rapid motor transfer and Spatial translation in the oculomotor system. Vision Res 124:1–6. 10.1016/j.visres.2016.06.002 27317977 10.1016/j.visres.2016.06.002

[CR50] Unsworth N, Schrock JC, Engle RW (2004) Working memory capacity and the antisaccade task: individual differences in voluntary saccade control. J Exp Psychol Learn Mem Cogn 30:1302–1321. 10.1037/0278-7393.30.6.1302 15521806 10.1037/0278-7393.30.6.1302

[CR51] Valdez P (2019) Circadian rhythms in attention. Yale J Biol Med 92:81–92 30923475 PMC6430172

[CR52] Valdez P, Ramírez, García A (2012) Circadian rhythms in cognitive performance: implications for neuropsychological assessment. ChronoPhysiol Therapy 2:81–92. 10.2147/cpt.S32586

[CR53] Van der Stigchel S, Hessels RS, van Elst JC, Kemner C (2017) The disengagement of visual attention in the gap paradigm across adolescence. Exp Brain Res 235:3585–3592. 10.1007/s00221-017-5085-2 28884226 10.1007/s00221-017-5085-2 PMC5671527

[CR54] Verbruggen F, Aron AR, Band GPH et al (2019) A consensus guide to capturing the ability to inhibit actions and impulsive behaviors in the stop-signal task. eLife 8:e46323. 10.7554/eLife.46323 31033438 10.7554/eLife.46323 PMC6533084

[CR55] Waterhouse J (2010) Circadian rhythms and cognition. Prog Brain Res 185:131–153. 10.1016/B978-0-444-53702-7.00008-7 21075237 10.1016/B978-0-444-53702-7.00008-7

[CR56] Wong-Lin K, Eckhoff P, Holmes P, Cohen JD (2010) Optimal performance in a countermanding saccade task. Brain Res 1318:178–187. 10.1016/j.brainres.2009.12.018 20034481 10.1016/j.brainres.2009.12.018 PMC2846395

